# The reverse transcriptase inhibitor 3TC protects against age‐related cognitive dysfunction

**DOI:** 10.1111/acel.13798

**Published:** 2023-03-22

**Authors:** Devin Wahl, Meghan E. Smith, Cali M. McEntee, Alyssa N. Cavalier, Shelby C. Osburn, Samuel D. Burke, Randy A. Grant, David Nerguizian, Daniel S. Lark, Christopher D. Link, Thomas J. LaRocca

**Affiliations:** ^1^ Department of Health and Exercise Science Colorado State University Fort Collins Colorado USA; ^2^ Center for Healthy Aging Colorado State University Fort Collins Colorado USA; ^3^ Department of Biochemistry and Molecular Genetics University of Colorado School of Medicine Aurora Colorado USA; ^4^ Department of Integrative Physiology University of Colorado Boulder Boulder Colorado USA

**Keywords:** Alzheimer's disease, brain aging, cognitive function, neuroinflammation, transcriptomics, transposable elements

## Abstract

Aging is the primary risk factor for most neurodegenerative diseases, including Alzheimer's disease. Major hallmarks of brain aging include neuroinflammation/immune activation and reduced neuronal health/function. These processes contribute to cognitive dysfunction (a key risk factor for Alzheimer's disease), but their upstream causes are incompletely understood. Age‐related increases in transposable element (TE) transcripts might contribute to reduced cognitive function with brain aging, as the reverse transcriptase inhibitor 3TC reduces inflammation in peripheral tissues and TE transcripts have been linked with tau pathology in Alzheimer's disease. However, the effects of 3TC on cognitive function with aging have not been investigated. Here, in support of a role for TE transcripts in brain aging/cognitive decline, we show that 3TC: (a) improves cognitive function and reduces neuroinflammation in old wild‐type mice; (b) preserves neuronal health with aging in mice and *Caenorhabditis elegans*; and (c) enhances cognitive function in a mouse model of tauopathy. We also provide insight on potential underlying mechanisms, as well as evidence of translational relevance for these observations by showing that TE transcripts accumulate with brain aging in humans, and that these age‐related increases intersect with those observed in Alzheimer's disease. Collectively, our results suggest that TE transcript accumulation during aging may contribute to cognitive decline and neurodegeneration, and that targeting these events with reverse transcriptase inhibitors like 3TC could be a viable therapeutic strategy.

## Graphical abstract

Age‐related increases in transposable element transcripts may contribute to brain aging and neurodegeneration by stimulating neuroinflammation. Targeting these transcripts with the reverse transcriptase inhibitor 3TC has the potential to improve cognitive function by reducing immune activation/neuroinflammation.

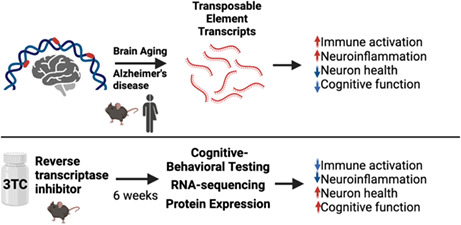

## 1 INTRODUCTION

Aging itself is the leading risk factor for neurodegeneration and Alzheimer's disease (Xia et al., 2018). Central mechanisms of brain aging include neuroinflammation/immune activation, declines in neuronal function/health and metabolic dysregulation (Mattson & Arumugam, 2018; Wahl et al., 2016), all of which contribute to reduced cognitive function with aging—a key risk factor for Alzheimer's disease (Wyss‐Coray, 2016; Yaffe et al., 2004). However, the specific upstream causes of these events remain incompletely understood.

One possible upstream and pro‐inflammatory mechanism of brain aging could be the dysregulation of transposable elements (TEs). These repetitive DNA sequences account for >50% of the human genome, and some have the ability to propagate and/or change locations in the genome (Bourque et al., 2018). Class 1 TEs (retrotransposons) mobilize via a “copy and paste” mechanism involving an RNA intermediate, which is reverse‐transcribed into cDNA that re‐integrates elsewhere in the genome. Class 1 TEs include Long Interspersed Nuclear Elements (LINEs), Short Interspersed Nuclear Elements (SINEs), and Long Terminal Repeats (LTRs). In contrast, class 2 TEs (DNA transposons) mobilize via a “cut and paste” mechanism with only a DNA intermediate (Bourque et al., 2018). The great majority of TEs are inactive (not transpositionally competent) and/or suppressed by heterochromatin, but some retrotransposons remain active in most organisms and are increasingly linked with aging and disease processes (Andrenacci et al., 2020; De Cecco et al., 2013; Gorbunova et al., 2021; Liu et al., 2023).

Importantly, we and others have reported progressive, age‐related increases in TE transcripts in model organisms and humans (LaRocca et al., 2020) and found that healthy aging interventions like calorie restriction, rapamycin, and exercise reduce TE transcripts in peripheral tissues (Wahl et al., 2021). Our data and others' point to a broad increase in most TE transcripts with aging, the most likely explanation for which is reduced genome‐wide chromatin maintenance, an established feature of aging (Andrenacci et al., 2020; Field & Adams, 2017; Ravel‐Godreuil et al., 2021; Sedivy et al., 2008). This global TE dysregulation could have many aging‐relevant consequences, but one in particular is that among the many TE transcripts that accumulate with aging, those from active retrotransposons have the potential to cause cellular inflammation, a hallmark of aging, as the cDNA they generate can stimulate innate immune sensors (Gorbunova et al., 2021). Consistent with this idea, the reverse transcriptase inhibitor 3TC has been reported to reduce peripheral inflammation in aging wild‐type mice (De Cecco et al., 2019), improve health and motor function in transgenic progeroid mice (Simon et al., 2019), and increase lifespan in *Drosophila* (Wood et al., 2016).

Despite good evidence of a link between age‐related TE dysregulation and inflammation in peripheral tissues, the role of TE transcripts in brain aging and the therapeutic potential of 3TC in this context are less clear. Recent data, although limited, suggest that other reverse transcriptase inhibitors might positively affect behavior in old mice (Liu et al., 2023). Others have also reported that transcripts from certain retrotransposons correlate with tau pathology (a key feature of Alzheimer's disease) (Guo et al., 2018), that inhibiting tau‐induced TEs with 3TC can protect against neurodegeneration in model organisms (Sun et al., 2018), and that tau may accelerate age‐related TE dysregulation in the mouse brain (Ramirez et al., 2022). However, the influence of reverse transcriptase inhibitors like 3TC on brain aging, cognitive decline, and neuroinflammation have not been comprehensively investigated, and whether TE dysregulation occurs in human brain aging per se is unknown (Ahmadi et al., 2020). Here, we report a series of experiments showing that 3TC: (1) crosses the blood–brain barrier, reduces neuroinflammation, and improves cognitive function in older wild‐type mice; (2) protects neuronal health/structure with aging, including in a model organism; and (3) enhances cognitive function and reduces anxiety responses in a mouse model of tauopathy. To demonstrate the clinical relevance of these observations, we also present bioinformatics analyses of existing human datasets showing that normal human brain aging (in the absence of pathology) is associated with increases in TE transcripts, and that these age‐related increases intersect with those observed in Alzheimer's disease.

## 2 RESULTS

### 2.1 3TC improves cognitive function in older mice

**FIGURE 1:**
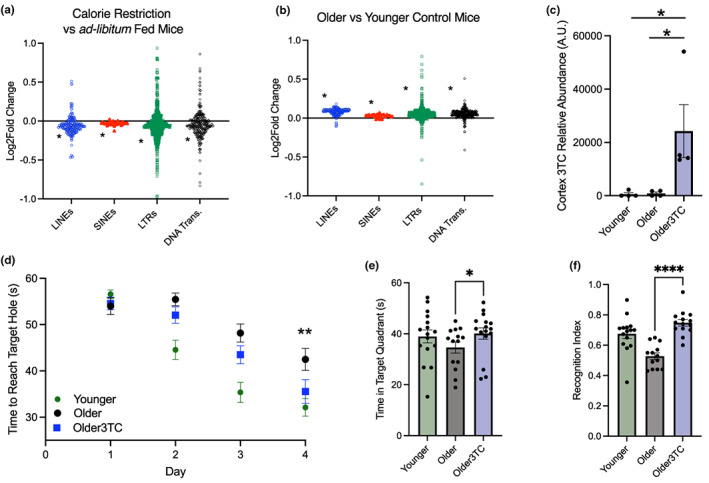
3TC improves cognitive function in older mice. (a) Re‐analysis of published RNA‐seq data showing TE transcript decreases in hippocampus of calorie‐restricted versus ad libitum‐fed mice. (b) Newly generated RNA‐seq data showing increases in TE transcripts in hippocampi of older versus younger mice in the current study. (c) Mass spectrometry analysis of 3TC in cortex of mice treated with/without 3TC for 6 weeks in drinking water. (d) Spatial memory acquisition in younger, older and older 3TC‐treated mice in the Barnes Maze, quantified as time to reach the target hole. (e) Probe trial (mean time spent in target quadrant). (f) Novel object recognition index quantified as time exploring the novel object versus total object exploration time. Younger mice, 9 mo; older mice, 20 mo. 3TC analysis, *n* = 4 mice/group; cognitive testing *n* = 14–16/group; RNA‐seq, *n* = 6/group. *p < 0.05; **p < 0.01; ****p < 0.0001. TE type comparisons: Shapiro–Wilk normality test followed by Mann–Whitney test versus mean Log2 fold‐change for all transcripts. 3TC, Novel Object Recognition, Probe Trial: ANOVA with Tukey's multiple comparison post‐hoc analysis; Barnes Maze: 2 × 2 mixed effects (day × treatment) repeated measures ANOVA.

Recent data demonstrate widespread TE transcript increases in the aging mouse brain that are accelerated by tauopathy (Ramirez et al., 2022). Because these broad increases included TEs with the potential to stimulate inflammation via the generation of cDNA (i.e., autonomously active mouse retrotransposons like L1 and IAP), we reasoned that inhibiting these events might improve cognitive function with aging. For proof of concept, we first analyzed TEs in existing brain RNA‐seq data on mice subjected to 20% calorie restriction (Wahl et al., 2018), perhaps the strongest intervention for improving brain health and cognitive function in mice (Wahl et al., 2021), using established bioinformatics pipelines as previously described (Criscione et al., 2014; Jin & Hammell, 2018; LaRocca et al., 2020; Wahl et al., 2021). We found that mice subjected to long‐term calorie restriction had lower levels of TE transcripts, including L1 and IAP elements, compared to ad libitum‐fed controls (Figure 1a and Supplementary Data). Given these findings in the hippocampus, which is centrally involved in memory/cognitive function (Mancini et al., 2022), and many reports that calorie restriction improves cognitive function and reduces neuroinflammation (Mattson, 2010), we next tested the idea that 3TC might also improve cognitive function. We provided older (aged 19 months) male and female C57Bl/6J mice with drinking water supplemented with or without 3TC (2 mg/mL) for 6 weeks. This dose of 3TC has been used in prior studies (De Cecco et al., 2019) and is reportedly similar to the human therapeutic dose (300 mg/day) (Else et al., 2012). Younger (8 months) male and female mice, as a reference group, received untreated drinking water. We did not note any changes in water intake, food consumption, or body weights in treated versus control mice, and 3TC was well‐tolerated with no clear adverse effects (Supplementary Figure 1a).

After the treatment period, we performed total RNA‐seq (to capture as many different transcript types as possible) on hippocampus samples from these mice, and we analyzed TE transcripts as described above. Although no individual TEs were significantly increased (perhaps because the age difference between older and younger mice in our study was less than in others', and/or because we analyzed a different brain region), we observed significant age‐related increases by group in all major types of TE transcripts, an effect that was not due to overall transcriptome differences, as gene counts/fold‐changes were evenly distributed (Figure 1b and Supplementary Figure 1b). In treated animals, mass spectrometry confirmed that 3TC crossed the blood–brain barrier (Figure 1c), similar to what others have reported in pre‐clinical models (Wu et al., 1998), and we observed strong effects of 3TC on cognitive function when we measured spatial memory acquisition and short‐term recognition memory via the Barnes Maze and Novel Object Recognition tests, respectively. Consistent with other reports (Cárdenas‐Tueme et al., 2022), in the Barnes Maze, older control mice were slower to find the escape hole over time/repeated trials versus young controls. In contrast, older 3TC‐treated mice performed similarly to younger mice (Figure 1d). These results were confirmed with a probe trial (24 h after final testing) in which the escape hole was removed and mice were allowed to explore for 1 min. Older 3TC‐treated mice spent more time in the target quadrant compared to older control mice, and a similar amount of time in the target quadrant compared to younger animals (Figure 1e). In the Novel Object Recognition test, a 2‐h inter‐trial interval was used to assess short‐term recognition memory, and we found that older 3TC‐treated mice performed significantly better than older control mice and similarly to younger mice as measured by recognition index (Figure 1f). These data show that 3TC improves cognitive function in aging non‐transgenic mice, which do not develop Alzheimer's disease‐related pathologies (consistent with the idea of a role for TE transcripts in brain aging per se).

### 2.2 3TC modulates the brain transcriptome

**FIGURE 2:**
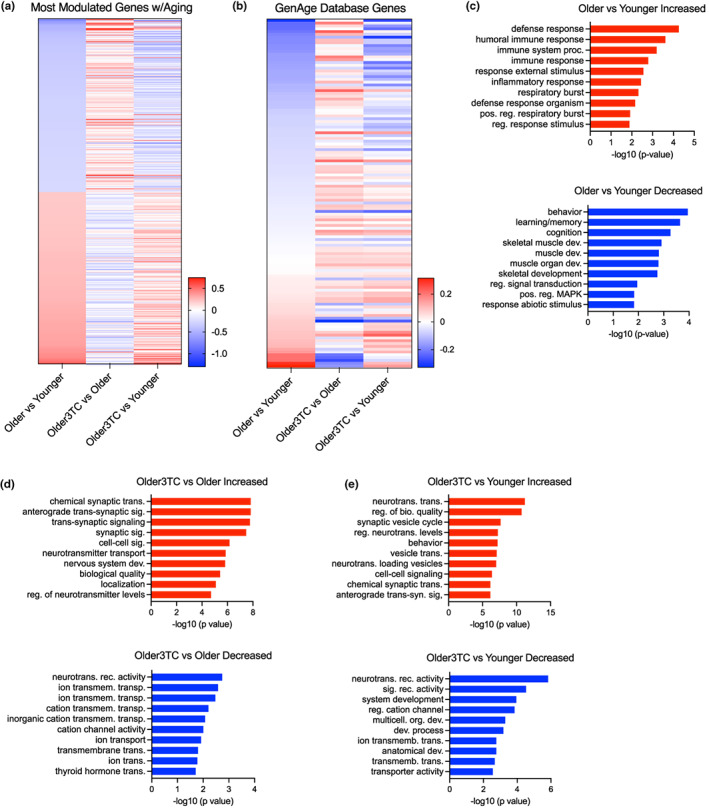
3TC influences gene expression patterns in the hippocampus. (a) Heatmap showing top 500 most significantly increased and decreased genes/transcripts with aging in the hippocampus of treated/untreated and younger/older mice. (b) Heatmap of genes reported to influence aging in the GenAge database (in the same groups of mice). (c–e) Increased and decreased biological processes (gene ontology [GO] terms) associated with overall transcriptome patterns in the same mice. All analyses based on RNA‐seq data from *n* = 6 mice/group. Parallel KEGG analyses shown in Supplementary Figure 2.

Many biological processes that mediate brain aging and impact cognitive function are reflected in the transcriptome (Ham & Lee, 2020). In our RNA‐seq data on treated and control mice, aging was associated with broad gene expression changes in the hippocampus (left and right heatmap panels, Figure 2a), but these effects were largely reduced with 3TC (Figure 2a, middle heatmap panel). In fact, ~85% of the top 500 increased and decreased genes/transcripts were reversed with 3TC treatment in terms of relative expression versus controls. Genes associated with aging and modulated by 3TC included Toll Like Receptor 7 (*TLR7*; increased with aging, decreased with 3TC), which is involved in innate immune responses (Shaw et al., 2011), and activity‐regulated cytoskeleton‐associated protein (*ARC*, decreased with aging, increased with 3TC), a regulator of synaptic plasticity involved in learning and memory (Fila et al., 2021). Because aging is the major risk factor neurogenerative diseases, we next examined age‐ and 3TC‐related effects on the expression of genes reported to influence lifespan in the GenAge database (Tacutu et al., 2018) (Figure 2b). Again, we found that most of the age‐related differences in expression of these genes trended in the opposite direction with 3TC (Figure 2b). Of note, one of the top genes downregulated with aging but ameliorated by 3TC was superoxide dismutase 3 (*SOD3*), which is centrally involved in preventing oxidative stress and neuroinflammation (Clausen et al., 2010). Another transcript reduced with aging and modestly modulated by 3TC was apolipoprotein E (*APOE*), which plays an important role in synaptic plasticity, cognitive function, and AD risk (Lane‐Donovan et al., 2016).

To identify biological processes associated with the age‐ and 3TC‐related transcriptome differences we observed in the brain, we performed Gene Ontology and KEGG analyses. In older versus younger mice, increased Gene Ontology and KEGG processes comprised pathways commonly implicated in brain aging including inflammation, immune responses, and neuronal function, whereas decreased biological processes were related to cognition, learning/memory, behavior, and synaptic signaling (Figure 2c and Supplementary Figure 2a). These findings are consistent with other reports on transcriptomic changes with brain aging in humans and model organisms (Cavalier et al., 2021; Loerch et al., 2008). In older 3TC‐treated mice versus older controls, increased biological processes included synaptic transmission and neurotransmitter communication, and decreased processes included ion transport, neurotransmitter receptor activity, and cytokine/MAPK signaling pathways—that is, the opposite of differences observed in older versus younger controls (Figure 2d and Supplementary Figure 2). Collectively, these results indicate that 3TC treatment reverses several biological and molecular processes related to brain aging and cognitive dysfunction, particularly neuroinflammation and immune response‐related pathways. Interestingly, healthy aging interventions like calorie restriction and intermittent fasting reverse similar adverse processes in the aging brain (Swindell, 2009), suggesting that targeting TE dysregulation with 3TC may be a novel strategy for promoting healthy brain aging.

### 2.3 3TC reduces inflammation and modulates metabolite signatures in the brain

**FIGURE 3:**
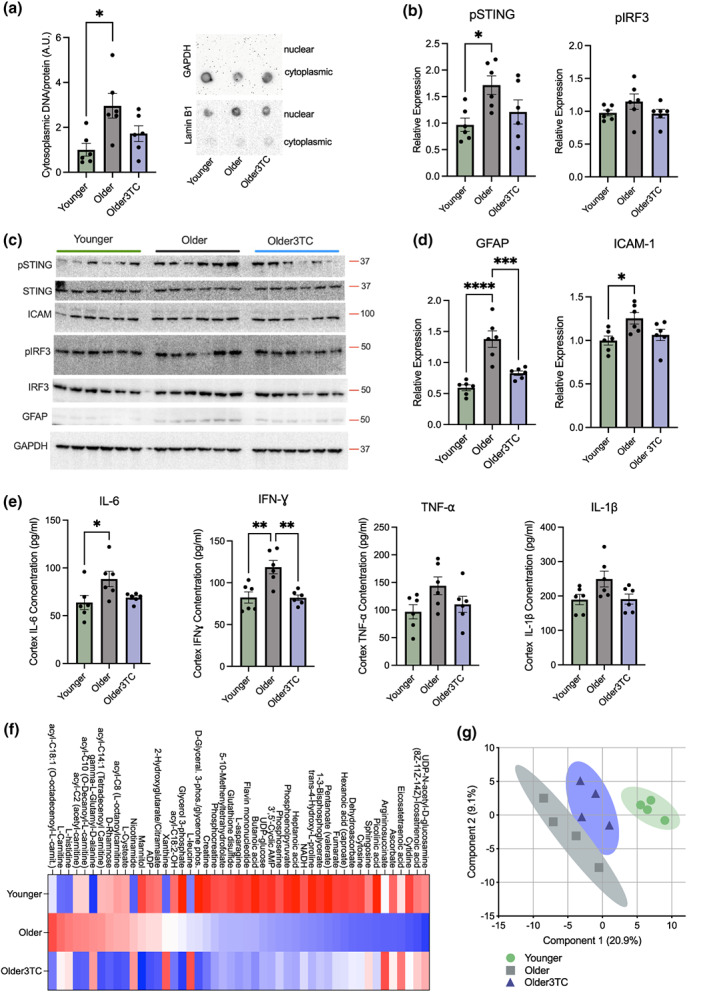
3TC influences neuroinflammation and metabolism in the brain. (a) Relative DNA concentrations in cytoplasmic fractions isolated from cortex of younger, older and older 3TC‐treated mice; representative dot blots confirming cytoplasmic (GAPDH) and nuclear (Lamin B1) fractions. (b–d) Western blot analyses of phosphorylated STING and IRF3, and GFAP and ICAM‐1 in the same mice. (e) Cortex concentrations of IL‐6, IFN‐ɣ, TNF‐α, and IL‐1β in the same mice detected by ELISA. (f) Cortex metabolite differences among groups measured by targeted mass spectrometry. (g) Partial least squares‐discriminant analysis of global metabolite variation among groups (shaded regions, 95% confidence intervals). ELISAs, immunoblotting: ANOVA with Tukey's multiple comparison post‐hoc analysis, *n* = 6/group; metabolomics, *n* = 4/group. *p < 0.05; **p < 0.01.

As a reverse transcriptase inhibitor, 3TC is presumed to protect against TE‐related aging phenotypes by limiting cDNA produced during reverse transcription of TE transcripts, and thus reducing activation of innate immune sensors like Cyclic GMP‐AMP synthase (cGAS) (De Cecco et al., 2019). Consistent with this idea, our RNA‐seq analyses suggested that major effects of aging reversed by 3TC included neuroinflammation and immune responses. We also found that cytoplasmic DNA levels were modestly increased in the brains of older mice, similarly to what others have reported with aging/inflammation (Lan et al., 2019; Miller et al., 2021), and that this effect was less pronounced in 3TC‐treated animals (Figure 3a, *p* = 0.11 vs. older controls). Therefore, we probed for cytoplasmic DNA sensors like cGAS and other proteins involved in immune activation and cellular responses to episomal DNA by immunoblotting (Figure 3b,c and Supplementary Figure 3a). We were unable to detect differences among groups in cGAS and activated (phosphorylated) cGAS, or in other cDNA sensors including Mov10 RISC Complex RNA Helicase (MOV10) and Three‐prime Repair Exonuclease 1 (TREX1) (data not shown). However, we did observe age‐related increases in activation (phosphorylation) of Stimulator of Interferon Genes (STING), a key mediator of cGAS signaling and the innate immune response to cDNA (van de Stolpe & van der Saag, 1996), whereas phosphorylated STING levels in 3TC‐treated old mice were similar to those observed in young animals (*p* = 0.09 vs. older controls). Similar but non‐significant trends were present for phosphorylated Interferon Response Factor 3 (IRF3), a transcriptional regulator of cGAS/STING responses that stimulates the production of type‐1 interferons and other inflammatory mediators (Decout et al., 2021). Furthermore, consistent with these age‐ and treatment‐related differences in cDNA/innate immune sensors, we observed age‐related increases that were absent with 3TC in Glial Fibrillary Acidic Protein (GFAP) and Intercellular Adhesion Molecule 1 (ICAM‐1) (Figure 3d), both of which are related to brain inflammation, glial cell (astrocyte) activation, and cognitive dysfunction (Chatterjee et al., 2021). Interestingly though, Allograft Inflammatory Factor 1 (Iba1), a marker of immune‐activated microglia, was unchanged across groups (Supplementary Figure 3a). Although modest, these effects of 3TC on neuroinflammation and innate immune markers suggest that the relatively small amounts of the drug that cross the blood brain barrier may have a limited but biologically relevant ability to reduce activation of cDNA sensing/immune activation pathways in the aging brain, perhaps by reducing TE‐derived cDNA. Future studies will be needed to confirm this idea, as our analyses were limited only to total cytoplasmic DNA and protein measurements of cDNA sensors (i.e., as opposed to their specific binding targets).

To further confirm the anti‐inflammatory effects of 3TC, we next measured downstream mediators of immune responses/inflammation via ELISA. We found that aging increased cortex concentrations of Interleukin 6 (IL‐6) and Interferon Gamma (IFN‐ɣ), pro‐inflammatory cytokines centrally involved in host defense and immune responses, including to retrotransposons (Castro et al., 2018; Custodero et al., 2022; Saleh et al., 2019). However, this effect was attenuated with 3TC treatment (Figure 3e). We were unable to detect changes among groups in levels of Tumor Necrosis Factor α (TNF‐α), Interleukin 1β (IL‐1β), or Interferon β (IFN‐β, which is more directly related to the cGAS/STING type‐1 interferon response), but there were non‐significant trends for age‐related increases and reversal by 3TC in several of these proteins (Figure 3e and Supplementary Figure 3b). We also noted that many of the top Gene Ontology and KEGG processes identified above (i.e., in Figure 2 and Supplementary Figure 2) were driven in part by increases in type‐1 interferon‐associated genes (e.g., IFNA5, IFNA1, IFNAR1, IFNAR2, IFNA14, and others, Supplementary Data). Finally, because peripheral inflammation has been linked to brain aging and inflammation (Corlier et al., 2018), we measured levels of TNF‐α, IL‐1β, IL‐6, and IFN‐ɣ in plasma from treated and control mice; again, we observed trends for differences in these cytokines with aging and 3TC, but these effects did not reach statistical significance (Supplementary Figure 3b). Thus, our results show that treatment with 3TC attenuates age‐related increases in some key mediators of inflammation and immune responses in the brain, which may partly explain its protective effects on cognitive function.

Importantly, inflammation/immune responses are associated with changes in energy and redox metabolism (Ye & Keller, 2010), and related changes in the metabolome are reportedly linked with Alzheimer's disease (Kim et al., 2014). Reverse transcriptase inhibitors like 3TC may also have adverse metabolic effects in peripheral tissues (Thet & Siritientong, 2020). We therefore sought to gain additional insight on the effects of 3TC in the brain by performing a targeted metabolomics study (~280 common metabolites) on cortex tissue. Although most effects were modest, we observed a partial normalization of age‐related changes in metabolites with 3TC, driven in part by modulation of acylcarnitines (involved in mitochondrial fatty acid metabolism) and metabolites of arachidonic acid (e.g., eicosatrienoic acids), which are linked with inflammatory signaling (Figure 3f). Partial least squares‐discriminant analyses to identify broader metabolite patterns showed clearer effects of age and 3TC treatment, with metabolite signatures of younger mice clustering near those of older 3TC‐treated mice (Figure 3g). Interestingly, pathway analyses (Supplementary Figure 3c) indicated that the broader metabolic pathways modulated most by both aging and 3TC included the metabolism of purines, nicotinamide, and several amino acids. These observations are consistent with the fact that 3TC inhibits reverse transcription because it is a nucleoside analogue and may, therefore, affect related metabolic pathways in the brain. However, these results could also suggest novel anti‐aging effects of 3TC via the modulation of metabolic pathways that change with aging, as nicotinamide/mitochondrial metabolites and several of the amino acids identified in these pathway analyses are involved in aging and lifespan (e.g., arginine, cystine, and methionine) (Trautman et al., 2022). Due to the targeted metabolomics approach we used, we could not draw more definitive conclusions from these data, but our results suggest that the beneficial effects of 3TC on cognitive function and neuroinflammation in older mice may involve at least a partial normalization of metabolic processes.

### 2.4 3TC protects neuronal health and structure with aging

**FIGURE 4:**
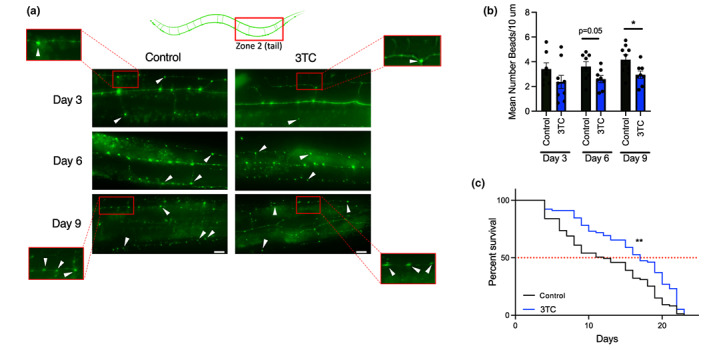
3TC reduces neurodegeneration and increases lifespan. (a) Representative images of PVD neurons in control and 3TC‐treated *Caenorhabditis elegans* during aging. Images of animal posterior (red box, top); white triangles represent dendritic “beads” that were quantified as a measure of neurodegeneration. White scale bar = 10 μm. (b) Mean number of dendritic beads/10 μm. *N* = 8–10 animals/group/day. (c) Survival curves for control and 3TC‐treated animals (*n* = 70–80 worms/group). *p < 0.05; **p < 0.01. Dendritic beading analyses: Student's *t*‐test; median lifespan comparisons: Log‐rank (Mantel‐Cox) test.

In addition to beneficial effects on neuroinflammation, we observed gene expression changes indicative of improved neuronal health with 3TC treatment (e.g., neurotransmitter transport and synaptic signaling), suggesting the drug may have inhibited neurodegeneration in mice. Neurodegeneration is characterized by a progressive loss of neurons and their axons/dendrites, which is associated with neuronal and cognitive dysfunction (Salvadores et al., 2017). We recently reported that TE transcripts, including from retrotransposons, are broadly increased with aging in *Caenorhabditis elegans* (LaRocca et al., 2020), which is often used as a model organism for studying neurodegeneration (Van Pelt & Truttmann, 2020). Therefore, to confirm the effects of 3TC on neuronal health and structure with aging, we employed a transgenic *C. elegans* strain expressing GFP in the PVD neuron (a main neuron involved in touch and sensory perception) (Tao et al., 2019). Similar to what others have reported (Lezi et al., 2018), we observed increases in “bead‐like” structures reflecting neurodegeneration along PVD dendrites with aging in these animals (Figure 4a). However, this effect was attenuated with 10 μM 3TC supplementation in food (Figure 4b). Also, consistent with other studies in *Drosophila* (Sun et al., 2018; Wood et al., 2016) and the primary role of aging in neurodegeneration (Culig et al., 2022), we found that these neuroprotective effects of 3TC were associated with an ~23% increase in median lifespan (Figure 4c). These results provide further evidence that 3TC may directly protect against neuronal aging/degeneration.

### 2.5 3TC improves cognitive function in a mouse model of tauopathy

**FIGURE 5:**
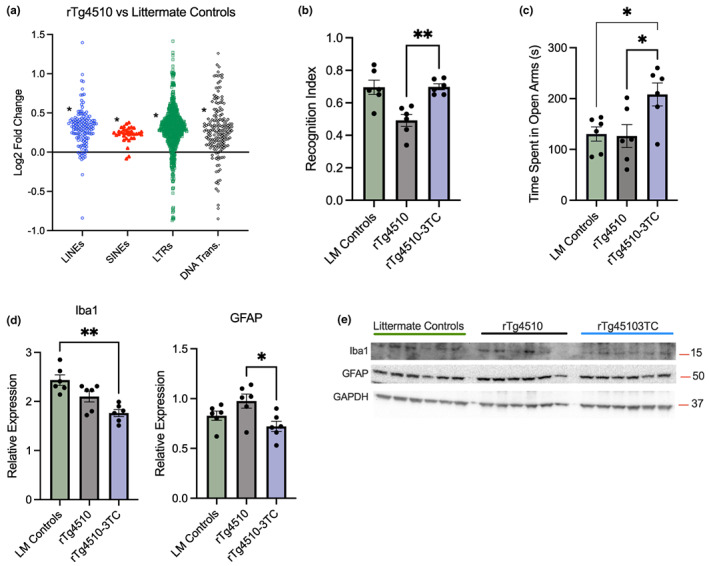
3TC improves cognitive function in a mouse model of tauopathy. (a) Re‐analysis of published RNA‐seq data showing TE transcript increases in the brains of 8‐month rTg4510 transgenic tauopathy mice versus littermate controls. (b) Novel Object Recognition index in littermate controls and rTg4510 mice treated with/without 3TC in the present study (quantified as time exploring the novel object vs. total object exploration, *n* = 6 mice/group; 3 months old). (c) Anxiety responses in the same mice as measured by time spent on open arms in the Elevated Plus Maze. (d, e) Immunoblot quantifications and images of Iba1 and GFAP. TE type comparisons: Shapiro–Wilk normality test followed by Mann–Whitney test versus mean Log2 fold‐change for all transcripts. Novel Object Recognition, Elevated Plus Maze and immunoblot analyses: ANOVA with Tukey's multiple comparison post‐hoc analysis. *p < 0.05; **p < 0.01.

Retrotransposon transcript increases have been linked to important hallmarks of Alzheimer's disease, most notably pathogenic tau, and this could be another mechanism by which TE‐associated cognitive impairments develop (Guo et al., 2018; Ramirez et al., 2022; Sun et al., 2018). As preliminary proof of concept for 3TC as a potential therapeutic in this context, we first analyzed a pre‐existing RNA‐seq dataset on cortex tissue from rTg4510 mice (a transgenic model of tauopathy due to the expression of mutant [P301L] human tau) (Castanho et al., 2020). In transcriptome data from the original study, tauopathy was associated with increased Gene Ontology evidence of inflammation/immune activation and decreases in signatures of neuronal health and function (much like in our older vs. younger mice), suggesting these mice could be a good model for testing the efficacy of 3TC—although we note that others have reported retrotransposon increases in other tau models as well (Ramirez et al., 2022). Consistent with previous reports and our observations in humans and mice, we found broad increases in TE transcripts, including individual retrotransposons and L1/IAP elements, in tauopathy versus littermate control animals by 8 months of age (Figure 5a and Supplementary Data), and similar trends were apparent earlier (at 4 months, Supplementary Data). Based on these observations, we treated younger (2 months old, prior to overt pathology to increase the chances of a therapeutic effect) male and female rTg4510 mice for 6 weeks with 3TC, and we measured short‐term recognition memory via Novel Object Recognition after the treatment. We found that 3TC‐treated tauopathy mice performed significantly better than their untreated counterparts and similarly to littermate controls (Figure 5b). As anxiety is often comorbid with dementia (Badrakalimuthu & Tarbuck, 2012), we also tested anxiety responses in the same mice via the Elevated Plus Maze, and we found that 3TC‐treated mice spent significantly more time in open arms of the maze (reflecting reduced anxiety) versus untreated mice and even littermate controls (Figure 5c). In this small pilot study, we did not perform comprehensive profiling of inflammatory markers. However, we did probe for several key proteins involved in neuroinflammation and cognitive dysfunction. Although tauopathy mice at this age did not have increases in Iba1 and GFAP (markers of glia‐associated neuroinflammation) relative to littermate controls, we found that 3TC modestly reduced these proteins in treated transgenic animals (Figure 5d,e). Together, these results suggest that in addition to improving cognitive function in normal aging, 3TC may have the potential to exert similar, protective effects against tauopathy.

### 2.6 TE transcripts increase in human brain aging

**FIGURE 6:**
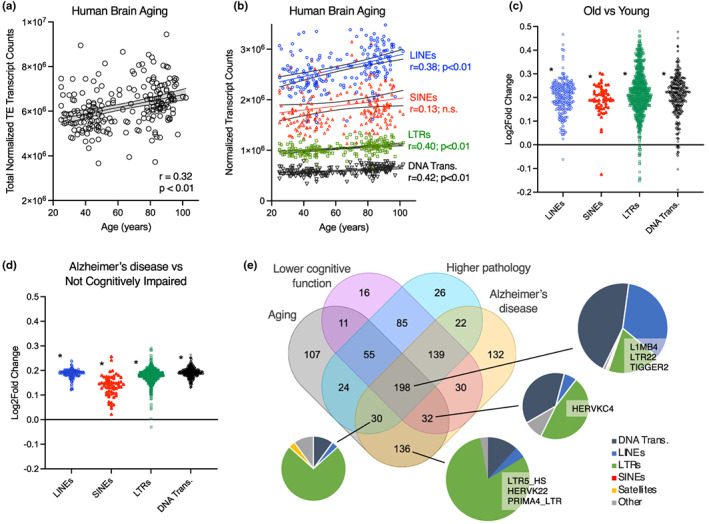
TE transcript accumulation in human brain aging and Alzheimer's disease. (a, b) Univariate correlations between age, total normalized TE counts, and TE counts by major type in RNA‐seq data on human brains in the NABEC dataset (*n* = 208, ages 25–102). (c, d) Plots showing increases in major TE transcript types in older versus younger brains (NABEC dataset), and in Alzheimer's disease patients (ROSMAP dataset) versus age/sex‐matched healthy controls (*n* = 10/group). (e) Overlap of individual TE transcripts most increased with aging and those most increased (>mean Log2 fold‐change) in all ROSMAP subjects with available data (*n* = 327 total) with lower cognitive function (lower 50% Mini Mental State Exams), higher pathology (highest 50% Braak staging) and Alzheimer's disease diagnosis, including TEs previously reported as tau/disease‐related. Correlations: Pearson correlations on normalized TE counts derived from DESeq2. TE type mean comparisons: Shapiro–Wilk normality test followed by Mann–Whitney test versus mean Log2 fold‐change for all transcripts.

To determine whether our findings in mice might be relevant in humans, we analyzed TEs in RNA‐seq data in the North American Brain Expression Consortium (NABEC) and Religious Order Study/Memory Aging Project (ROSMAP) datasets (De Jager et al., 2018; Dillman et al., 2017; Mostafavi et al., 2018) as described above. We found a positive correlation between age and TE transcript counts in the NABEC dataset (Figure 6a), and when we examined the most common TE types we found a similar relationship (Figure 6b). These age/TE correlations remained mostly significant (*p* < 0.05 for TEs by type, except SINEs; *p* = 0.11 for total TEs) after correcting for sex and post‐mortem interval. However, to examine age‐related TE transcript increases more carefully, we performed a matched comparison of 10 old and 10 young subjects (to reduce potential inter‐individual/sample variability effects) (Figure 6c and Supplementary Data). This analysis revealed a similarly broad, age‐related increase in TE transcripts, which was not due to differences in overall transcript levels (Supplementary Figure 4a). Interestingly, individual TEs that increased with aging did not include transpositionally competent LINE‐1 elements like L1HS. However, multiple HERVKs, including the HERVK HML‐2 family sequences HERVK‐int and LTR5_HS, were increased with aging (FDR ~ 0.05). These sequences reflect HERVK elements encoding reverse transcriptases (Garcia‐Montojo et al., 2018; Subramanian et al., 2011), which were recently reported to be increased with aging but targetable with reverse transcriptase inhibitors in other cells/tissues, and to play a key role in senescence (Liu et al., 2023). Importantly, these age‐related TE transcript increases were also associated with gene expression signatures indicating increased neuroinflammation and decreased neuronal health/function (Supplementary Figure 4b,c). These findings are in agreement with what we and others have reported as transcriptomic signatures of brain aging (Cavalier et al., 2021; Ham & Lee, 2020; Nativio et al., 2018), and with what we observed in aging mice (i.e., in Figure 2). Next, using brain RNA‐seq data from the ROSMAP study, we analyzed TE transcripts in Alzheimer's disease patients and cognitively normal controls. Similar to aging, we found an increase in all types of TE transcripts in a carefully sex‐ and age‐matched comparison of Alzheimer's disease patients versus controls (Figure 6d). Increased TE transcripts in these comparisons included many retrotransposons, and this again was not due to overall transcriptome differences (Supplementary Figure 4d). These results are consistent with previous reports (Guo et al., 2018; Saldi et al., 2019; Sun et al., 2018), including the observation that age‐ and disease‐associated TE transcript increases are broad, non‐specifically affecting most/all TEs, and we have observed similar effects of age and disease in other datasets—including ROSMAP and some in which age/Alzheimer's disease‐related chromatin dysregulation has been reported (e.g., in Nativio et al., 2020). Alzheimer's disease gene expression signatures in our analyses also reflected key hallmarks of neurodegenerative disease (Burgaletto et al., 2020) including increased neuroinflammation and immune responses (Supplementary Figure 4e,f).

Finally, given previous findings linking tau with TE activation in Alzheimer's disease (Guo et al., 2018; Sun et al., 2018), our finding that TE transcript levels increase with aging suggested that age‐ and pathology‐related TE dysregulation might intersect. To investigate this possibility, we examined patterns among the most commonly dysregulated individual TEs by intersecting age‐related TE transcripts with those most increased in subjects with higher pathology, lower cognitive function, and Alzheimer's disease in the ROSMAP dataset (Figure 6e, differential expression analyses by group). Consistent with existing data, in this comparison we found a large number of TEs (139 and 22) that were mostly increased with pathology and disease (but not aging), including several that others have reported to correlate with tau pathology (e.g., L1MB4). However, 198 of the age‐related TE transcripts we identified in the NABEC dataset were also associated with pathology, reduced cognitive function and disease, and these included some TEs previously identified as tau/disease‐related (e.g., HERVKC4 and PRIMA4). Furthermore, we found that many age‐related TEs were associated with lower cognitive function and/or Alzheimer's disease but *not* pathology; most of these were retrotransposons, especially LTRs, and they included the HERVK‐related sequences LTR5_HS and LTR13 (Subramanian et al., 2011). Collectively, these observations support the idea that brain aging is associated with a broad dysregulation of TE transcripts, including numerous retrotransposons that could have deleterious effects, and that these events may be: (1) further exacerbated in Alzheimer's disease, perhaps by tau as others have reported in mice (Ramirez et al., 2022); and (2) involved in age‐associated cognitive decline and neurodegeneration independent of pathology.

## 3 DISCUSSION

Brain aging, characterized by declines in cognitive function, is a major driver of many neurodegenerative diseases and dementias (Le Couteur & Thillainadesan, 2022). As such, there is an urgent need to identify interventions that can delay or reverse brain aging and preserve cognitive function. In this study, we demonstrate that the reverse transcriptase inhibitor 3TC, which has previously been shown to target TEs/retrotransposons in peripheral tissues, improves cognitive function in aging mice and a mouse model of tauopathy. We also provide evidence of biological mechanisms by which 3TC may protect cognitive function (e.g., by reducing activation of cDNA sensors, neuroinflammation/immune activation, and protecting neuronal health), and we show that TE transcripts accumulate with brain aging in humans. Our findings are clinically relevant, as TE transcripts may have potential as a biomarker of brain aging and Alzheimer's disease (Macciardi et al., 2022), and 3TC is a clinically translatable drug that has been FDA‐approved for over 25 years (Grodeck & Vazquez, 1997). In fact, several ongoing clinical trials (NCT04552795, NCT04500847 and NCT04993768) are assessing the safety and effects of 3TC and other reverse transcriptase inhibitors in Alzheimer's disease and other tauopathies (Macedo et al., 2022). As such, our data could provide a basis for similar studies earlier in the lifespan (i.e., to inhibit neuroinflammation, preserve cognitive function, and reduce disease risk before pathology develops).

Growing evidence implicates TE dysregulation in aging (De Cecco et al., 2019; Gorbunova et al., 2021; LaRocca et al., 2020; Liu et al., 2023), but most studies, including those testing the protective effects of 3TC in this context, have focused on peripheral tissues. Here, we provide evidence that TE dysregulation may play a role in brain aging independent of pathology. By analyzing existing data, we showed that calorie restriction, which strongly protects cognitive function in pre‐clinical models (Mattson, 2010), broadly reduces TE transcripts, including retrotransposons, in the hippocampus of older mice. We then performed a prospective study and showed that treating older mice with 3TC improves spatial and short‐term recognition memory. These findings are important because reduced cognitive function, especially in the domains of memory/learning, processing speed and executive function, is a key consequence of brain aging that directly increases the risk of chronic neurodegenerative disorders, including Alzheimer's disease (Fjell et al., 2017; Murman, 2015). Our results are also in line with other studies that have found functional improvements with 3TC treatment in aging mice. For example, 3TC has been shown to reduce anxiety and improve muscle function and bone density in the SIRT6 knockout mouse (a model of severe premature aging) (Simon et al., 2019). Another recent study demonstrated that 3TC improves spatial memory, reduces type‐1 interferon responses, and increases hippocampal neurogenesis in SAMP8 mice, a model of accelerated/senescence‐associated aging (Li et al., 2021), and new but limited data suggest reverse transcriptase inhibition might ameliorate behavior in old mice (Liu et al., 2023). Here, we expand upon these findings by showing that that 3TC crosses the blood–brain barrier and improves multiple domains of cognitive function in aging wild‐type mice, which do not develop tau‐related pathology, and we provide evidence of the mechanisms that may be involved, as described in the following sections.

First, to provide insight on the effects of 3TC in the brain in the present study, we performed transcriptomics (total RNA‐seq) on hippocampus tissue from mice subjected to long‐term 3TC treatment, which allowed us to examine both TE and gene expression. In these data, we found that TE transcript levels by major type were increased with aging, and this was independent of changes in overall gene/transcript expression—similar to our findings in human brain aging and Alzheimer's disease. No individual TEs were particularly increased in our data, which could be due to the ages of mice studied (less age difference between old and young mice in our study compared to others) and/or a result of the global TE dysregulation observed in aging and the underlying biology. For example, as reported by others, the most likely mechanism underlying TE transcript increases with aging and disease is epigenetic dysregulation (i.e., reduced maintenance of heterochromatin) (Field & Adams, 2017; Frost, 2016; Nativio et al., 2018). This would broadly/randomly affect the entire genome and differ among individual subjects and organisms. As such, differential expression analyses would likely reflect global TE dysregulation (i.e., with few or no individual transcripts being significantly altered versus others, but rather a broad increase in most). Total RNA‐seq data, like those generated here, might also spread reads across a greater number of TEs, adding further variability. These limitations of our approach may explain why we did not find significant increases in specific, active mouse TEs like IAP with brain aging, as others have reported (Ramirez et al., 2022). We also note that a limitation of short‐read RNA‐seq like we performed is that it cannot be used to confirm whether TEs are in protein‐coding genes, which could reflect another mechanism driving TE dysregulation (i.e., aberrant gene transcription with passenger TEs). Still, the general TE dysregulation we observed with aging in mice was associated with broad changes/patterns in hippocampal gene expression reflecting immune responses and neuroinflammation, consistent with our observations in human brains and other reports on brain aging (Cavalier et al., 2021; Dillman et al., 2017; Ham & Lee, 2020), and with a role for TE transcripts in inflammation. However, 3TC treatment reduced most of these age‐related transcriptome signatures in old mice. These findings agree with other studies on the influence of 3TC on inflammation and immune activation in peripheral tissues. For example, De Cecco et al. (2019) showed that LINE retrotransposons drive a type‐1 interferon response during aging, and that 2 weeks of 3TC treatment in drinking water reduced this response along with markers of age‐associated inflammation in peripheral tissues (e.g., kidney and muscle).

The presumed protective mechanism of action for 3TC is the inhibition of cDNA production via reverse transcription by active retrotransposons among the many TEs that increase with aging—which would reduce the activation of cytoplasmic cDNA/immune sensors. To investigate this in the present study, we probed for several mediators of immune activation and neuroinflammation. In old mouse brains, we found increases in activated (phosphorylated) STING, a central mediator of cDNA sensing and inflammation (Ou et al., 2021), but phosphorylated STING levels in 3TC‐treated older mice were similar to those observed in young animals. Similarly, we found that 3TC mitigated age‐related increases in GFAP and ICAM‐1, which are related to glial activation, neuroinflammation, and cognitive dysfunction (Chatterjee et al., 2021). We also found that 3TC modulated some pro‐inflammatory cytokines in the aging brain, including IFN‐ɣ and IL‐6 (modest reductions with 3TC treatment), both of which contribute to age‐related neuroinflammation and immune responses (Ramesh et al., 2013), and that all of these 3TC‐related changes were associated with somewhat lower levels of cytoplasmic DNA. Collectively, these findings support the idea that 3TC may have protected cognitive function by reducing retrotransposon‐derived cDNA, immune activation, and neuroinflammation. However, we cannot rule out other possibilities. For example, 3TC may have improved aging phenotypes by protecting against retrotransposition (i.e., re‐integration of retrotransposons into the genome, which can cause DNA damage and other adverse effects) (Bourque et al., 2018; Gorbunova et al., 2021; Saleh et al., 2019), especially in the brain where retrotransposon activity is particularly high (Terry & Devine, 2019). Indeed, other studies have shown that 3TC may reduce retrotransposon copy number, particularly of LINEs, in the genome (De Cecco et al., 2019; Simon et al., 2019), and this could in turn reduce transcript levels. We did not observe significant reductions in TE transcripts or in transcriptomic signatures of DNA damage responses with 3TC treatment, perhaps because small amounts of 3TC reach the brain or our treatment period was relatively short, but we also did not measure retrotransposon DNA copy number or cytoplasmic TE‐derived DNA specifically—and there is growing evidence for an accumulation of cytoplasmic DNA from multiple sources in aging (Lan et al., 2019; Miller et al., 2021). Thus, while the current results may be most consistent with other reports showing that inhibition of cDNA‐induced inflammation underlies the beneficial effects of 3TC in the brain, additional work is needed to confirm this and/or other mechanisms. For example, future studies could increase the expression of specific TEs in the brain and determine whether this causes aging/inflammation, as others have done in *Drosophila* and in cell culture (Liu et al., 2023; Rigal et al., 2022), or block specific TEs in the brain and determine whether 3TC is still effective (or if TE inhibition alone recapitulates its effects).

To explore other potential mechanisms in the present study, and because reverse transcriptase inhibitors may modulate metabolism (Note et al., 2003), we examined metabolites downstream of gene expression. In a targeted metabolomics analysis, we found that 3TC shifted the brain metabolome of old mice closer to that of young animals, in part by modulating metabolites related to inflammatory signaling. Interestingly, metabolic pathway analyses also showed that the pathways most modulated by aging and 3TC included the metabolism of purines, several amino acids, and mitochondrial metabolites (e.g., nicotinamide). These observations are consistent with the fact that 3TC is a nucleoside analogue, but their interaction with aging (and the fact that amino acid and mitochondrial metabolism are altered in aging) could also suggest other, possibly beneficial off‐target effects of the drug (e.g., mitochondrial hormesis). This possibility requires further study, as our analyses were limited and exploratory, and there are well‐known concerns about metabolic side effects of 3TC in peripheral tissues (Max & Sherer, 2000; Thet & Siritientong, 2020). Indeed, our data suggest that 3TC may exacerbate the brain aging phenotype for certain metabolites (e.g., l‐leucine, which is thought to contribute to insulin resistance, a driver of brain aging, and metabolic dysfunction in rodents) (Solon‐Biet et al., 2019), so this may be an important area for future work.

Importantly, in addition to neuroinflammation, we found that hippocampal aging in mice was associated with gene expression signatures reflecting reduced neuronal health, and that this was reversed by 3TC. Based on these observations, we used a model organism to determine whether 3TC may directly protect aging neurons. *C. elegans* PVD neurons serve as a model to study how dendritic morphology changes with aging (Yuval et al., 2021); here, we showed that the PVD neuron degenerates with aging, consistent with other reports that have linked this degeneration with immune sensor activation (Lezi et al., 2018), but that 3TC protects against this degeneration and preserves dendritic structure. Dendrites also degenerate with aging in most mammals (Furusawa & Emoto, 2021), and therefore, our results could suggest a neuron‐specific mechanism by which 3TC may improve cognitive function in mice. We also found that 3TC increased median lifespan in *C. elegans*, which may be unsurprising given the observation that neuronal health is closely related to longevity (Crimmins, 2015). To the best of our knowledge, reverse transcriptase inhibitors have not previously been studied for their effects on worm lifespan, and we note that retrotransposons are less common in the *C. elegans* genome than others. We also did not repeat these experiments in any mutant worm strains to rule out the possibility of hormesis‐related effects. However, our prior work (LaRocca et al., 2020) shows age‐related increases in most TE transcripts in the worm, including numerous CR1 and CER/Gypsy retrotransposons similar to those linked with lifespan in *Drosophila* (Wood et al., 2016), and 3TC has been shown to protect against neurodegeneration in *Drosophila* (Sun et al., 2018). Thus, our observations in non‐transgenic, aging *C. elegans* may be additional evidence of an interaction between neurodegeneration and age‐related TE dysregulation per se (i.e., as suggested by our data on human brains and others' data on mice) (Ramirez et al., 2022). These observations also further support the idea that a few active retrotransposons may be responsible and sufficient for the deleterious effects of global TE dysregulation, and that 3TC may act by reducing the overall burden of TE transcript activity below an immune‐sensing threshold in this context (Gorbunova et al., 2021).

Next, as preliminary proof of concept for the idea of targeting TE/retrotransposon transcripts in Alzheimer's disease, which is characterized by tau pathology (Wyss‐Coray, 2016; Xia et al., 2018), we analyzed an existing RNA‐seq dataset on rTg4510 tauopathy mice (Castanho et al., 2020). We found that TE transcripts were increased in these animals, similar to recent reports (Ramirez et al., 2022). Then, we prospectively treated rTg4510 mice with 3TC and found that it improved recognition memory and reduced anxiety. These findings are consistent with other studies demonstrating that elevated retrotransposon activity is associated with impairments in recognition memory in a mouse model of Down syndrome (also a tauopathy), and that 3TC may be protective in this context (Martinez de Lagran et al., 2022). In the present study, we also provided evidence that these effects of 3TC in tauopathy are associated with reductions in markers of activated glial cells, suggesting a role for neuroinflammation, which is implicated in both brain pathology and aging. These findings are more related to tau/TE interactions than to brain aging per se, but they provide further evidence that TE transcripts impair cognitive function, and additional support for the idea that reverse transcriptase inhibition may protect against these events (especially given that cognitive dysfunction and pathology are pronounced at the relatively late age of the rTg4510 mice studied here). We note that, as with many transgenic models of human neurodegeneration, there are potential controversies around the exact causes of tauopathy in rTg4510 mice (Yokoyama et al., 2022). As such, future studies in other transgenic mouse models are needed to determine whether similar mechanisms underlie tau‐ and age‐related TE dysregulation, and the extent to which the downstream consequences like neuroinflammation are similar, as this may provide insight on the utility of drugs like 3TC for treating aging versus disease.

Finally, we showed that TE transcripts also increase with human brain aging in the NABEC dataset (Dillman et al., 2017) and with Alzheimer's disease in the ROSMAP dataset (De Jager et al., 2018). Others have reported similar findings and correlated specific TE transcripts with tau pathology using ROSMAP data (Guo et al., 2018). However, to the best of our knowledge, the present analyses are the first to demonstrate age‐related increases in TE transcripts in the human brain—including at younger ages before tau pathology would be expected. Interestingly, TEs that increased with aging included HERVK elements, which have recently been shown to play a key role in aging/cellular senescence (Liu et al., 2023) and neurodegeneration (Dembny et al., 2020). Our data also suggest that this age‐related TE transcript accumulation intersects with TE dysregulation in Alzheimer's disease, which is consistent with recent data in mice (Ramirez et al., 2022). Similar to our findings in mice, we did not identify specific TE transcripts that drive brain aging and Alzheimer's disease, and we note that the tau/disease‐related TEs identified in different studies generally are not the same from one study to the next (Guo et al., 2018; Sun et al., 2018), consistent with the idea that non‐specific chromatin dysregulation may underlie these effects. Nevertheless, by intersecting age‐related TE increases with the most increased TE transcripts in large group comparisons of cognitive function, pathology and dementia, we found that some age‐related TE transcripts overlapped with those previously linked with tau pathology (Guo et al., 2018; Sun et al., 2018), whereas some (particularly from LINEs and LTRs) were more associated with brain aging/cognitive decline and disease. These results underscore: (1) the importance of the aging process itself in the dysregulation of TE transcripts in the brain (including potentially active retrotransposons that are likely the most deleterious among the many TEs dysregulated); and (2) the possibility that this dysregulation may contribute to brain aging and neurodegeneration independent of pathology (e.g., by increasing neuroinflammation/immune activation).

Collectively, our data add to the increasing evidence of a role for TE transcripts in aging and disease. Given this growing literature, determining the potential targetability of TE dysregulation with aging in different tissues is a key research goal. Our results suggest that TE dysregulation may be an important mechanism of brain aging, and that targeting TE transcript activity in this context may be a viable strategy for preserving cognitive function and perhaps preventing neurodegenerative disorders like Alzheimer's disease.

## 4 EXPERIMENTAL PROCEDURES

### 4.1 Animal husbandry

Younger (8 months) and older (19 months) male and female C57/B6 mice were purchased from the National Institute on Aging aged rodent colony. Male and Female rTg4510 mice and littermate controls (2 months of age) were purchased from the Jackson Laboratory. Mice were housed four per cage on a 12‐h light/dark cycle at 22–24°C at the laboratory animal facility at Colorado State University. All animals were given free access to water and food (Teklad Global Irradiated 18% protein rodent diet) and acclimatized to the facility before being randomly assigned to experimental groups. Body weights, food consumption, and water intake were measured every week, and all animals were routinely monitored by veterinary staff. All animal procedures were approved by Colorado State University, IACUC protocol #1441.

### 4.2 3TC treatment

3TC (National Drug Code: 33342‐002‐07, manufactured by Macleods Pharmaceuticals) was purchased from Health Warehouse and added directly to drinking water (2 mg/mL). Experimental cohorts were treated for 6 weeks with 3TC, and controls were provided with the same water but without the drug. Fresh water was routinely provided to all cages every 3–4 days.

### 4.3 Behavioral testing

Animals were handled extensively before testing to familiarize them with the experimenter and minimize anxiety. The same experimenter performed all behavioral tests. Mice received a minimum of 2 h room habituation before the start of each experiment and 2 days rest between different tests. Equipment was cleaned with 80% ethanol between tests to minimize scents. The Barnes Maze short‐term spatial memory test consisted of a white opaque circular table (90 cm in diameter) with 20 equally spaced holes around the perimeter. Spatial orientation cues (e.g., large poster triangle, squares) were placed on walls around the room and remained in the same position for the testing period. Animals underwent 4 trials per day, and if an animal was not able to find the escape hole after 1 min, it was gently guided to the hole and covered for 1 min. Novel Object Recognition tests were carried out as previously described (Chesworth et al., 2018). Mice were habituated for 5 min to the arena with two identical objects (tower of Legos) placed 8 cm from each wall. Following an inter‐trial interval of 2 h, mice were returned to the arena but one object was replaced with a novel object of similar shape, color, and size (a cell culture flask filled with multi‐colored sand). The testing trial ended once a total of 20 s exploration time was reached or 10 min had elapsed. The recognition index was calculated as the time each mouse spent exploring the novel object divided by total object exploration time. The Elevated Plus Maze test was performed as previously described (Okun et al., 2010). The maze consisted of a cross with four arms (67 cm in length; 2 exposed arms, 2 arms with high walls). Mice were placed in the center of the maze and allowed to freely explore for 5 min. Time spent in the open arms was recorded as a measure of anxiety. The Barnes Maze and Elevated Plus Maze studies were recorded and analyzed with behavior cloud software (behaviorcloud.com) to track and analyze mouse behavior and time spent in specific regions of each maze.

### 4.4 Animal sacrifice and tissue collection

Mice were culled in a fed state following 6 weeks of 3TC treatment in drinking water. All culls occurred in the late morning. After deep anesthetizing with isoflurane, approximately 1 mL of blood was removed via cardiac puncture; this was followed by cervical dislocation. Brains were quickly removed, and the full left hippocampus and a piece of left cortex tissue were carefully dissected, immediately flash‐frozen on dry ice and stored at −80°C until further processing. Corresponding right hippocampus and cortex sections were collected for immunoblotting, ELISAs and other biochemical analyses. For plasma isolation, blood was placed in an EDTA‐loaded microfuge tube and centrifuged for 15 min at 14,000 RPM.

### 4.5 RNA‐seq and transcriptome analyses on mouse tissue

RNA isolation and transcriptome analyses were performed as recently reported (LaRocca et al., 2019, 2021; Wahl et al., 2021). RNA was isolated from whole hippocampal sections using Zymo Direct‐zol Microprep kits according to manufacturer instructions. Total RNA libraries were generated using Illumina Ribo‐zero kits to remove ribosomal RNA, and libraries were sequenced on an Illumina NovaSeq 6000 platform to generate >40 M 150‐BP paired‐end reads per sample (Genomics Core, University of Colorado Anschutz Medical Campus). TE transcripts were analyzed with both the TEtranscripts (Jin & Hammell, 2018) and RepEnrich2 (Criscione et al., 2014) programs to confirm similar findings with different analysis pipelines as previously described (LaRocca et al., 2020). Reads were trimmed and filtered with the *fastp* program (Chen et al., 2018), then mapped to the mouse genome (mm10) with the STAR aligner (Dobin et al., 2013) with the following parameters which, as suggested in the TEtranscripts manual, yielded similar percentages of multi‐mappers and fewer significant TE hits (i.e., more conservative) in our analyses: ‐‐winAnchorMultimapNmax 200 ‐‐outFilterMultimapNmax 100. Gene counts from STAR and TE transcript counts (generated in TEtranscripts, which counts multi‐mapping reads against a repeatmasker annotation file with all TEs, including class 1 and 2 TEs, satellite sequences, tRNAs, etc.) were analyzed together for differential expression in R using DESeq2 software (Love et al., 2014), all mappable reads and the default Wald test. Normalized counts extracted from DESeq2 were used for all statistical analyses. Gene Ontology analyses (Biological Processes/KEGG pathways) were performed using g:Profiler (Raudvere et al., 2019).

### 4.6 Immunoblots and cytoplasmic DNA measurements

Cortex tissue was bead homogenized (Next Advance Bullet Blender) in ice‐cold RIPA buffer containing 100 mM KCl, 40 mM Tris HCl, 10 mM Tris base, 5 mM MgCl_2_, 1 mM EDTA, and 1 mM ATP (pH 7.5) with phosphatase and protease inhibitors (Thermo Fisher). Protein concentration was determined using a BCA kit (Thermo Fisher/Pierce). 20 μg of protein per sample was separated by electrophoresis (Bio‐Rad Criterion system) and transferred to nitrocellulose membranes (Trans‐Blot Turbo, Bio‐Rad) then blocked. Primary antibodies (all from ABclonal) included p‐IRF3 (1:1000 dilution), IRF3 (1:1000), p‐STING (1:1000), STING (1:1000), p‐cGAS (1:1000), cGAS (1:1000), IBA1 (1:750), pTBK1 (1:1000), TBK1 (1:1000), and GFAP (1:1000). GAPDH (1:2000, Novus Biologicals) was used for normalizing protein expression on all blots. Membranes were incubated in primary antibody overnight at 4°C followed by HRP‐conjugated secondary antibodies (1:5000; Cell Signaling) for 1 h at room temperature and then 1 min in ECL reagent (ThermoFisher/Pierce) for imaging on a ProteinSimple Alpha Imager. Cytoplasmic DNA was measured by lysing and fractionating remaining samples according to standardized protocols (Mosley & Baker, 2022; Spada et al., 2019) with a cytosolic‐nuclear fractionation kit optimized for frozen tissues (Invent Biotechnologies). Protein concentration was determined for each fraction using a BCA kit. Samples were diluted to equal concentrations, and 1 μg total protein/sample was spotted onto a nitrocellulose membrane in 2 μL total volume then dried, blocked, and incubated with primary antibodies overnight: GAPDH (1:1000) and Lamin B1 (1:1000, Cell Signaling). HRP‐conjugated secondary antibodies and ECL reagent were used for imaging as above. DNA concentrations were measured in diluted cytoplasmic fractions using a Quant‐iT PicoGreen high sensitivity dsDNA kit (ThermoFisher/Invitrogen) and a Qubit fluorimeter (ThermoFisher/Invitrogen) then normalized to GAPDH intensity for analyses.

### 4.7 ELISAs

Frozen cortex samples were homogenized in RIPA buffer (ThermoFisher) with fresh protease and phosphatase inhibitors (Roche, Sigma). Following centrifugation for 20 min at 14,000 RPM, supernatant was transferred to a fresh tube and protein concentrations were measured using a BCA assay (Thermo Scientific, Pierce). Cytokines were assessed in aliquots of lysate containing 40 μg of protein using commercially available kits as previously reported (Brunt et al., 2021) according to manufacturers' instructions: IL‐1β, TNF‐α, IL‐6, and IFN‐ɣ (R&D Biosystems); IFN‐β (PBL Assay Science). Levels of TNF‐α, IL‐6, IFN‐ɣ, and IL‐1β in plasma were measured by multiplex ELISA (Boster Biotech).

### 4.8 Metabolomics

Brain tissue samples were analyzed by ultra‐high pressure liquid chromatography coupled with mass spectrometry at the University of Colorado School of Medicine Metabolomics Core as previously described (D'Alessandro et al., 2021; Nemkov et al., 2021). Metabolites were manually annotated and integrated with Maven (Princeton University) in conjunction with the KEGG database. Characterization and assignment of 3TC in brain samples were performed against a commercial standard (Cayman Chemical Company). Peak quality was determined using blanks, technical mixes, and ^13^C abundance. Statistical analyses and pathway analyses were performed on normalized metabolite abundance data using established algorithms in the MetaboAnalyst 5.0 program (Pang et al., 2021).

### 4.9 *Caenorhabditis elegans* studies

The PVD::GFP *C. elegans* strain (NC1686) was purchased from the *Caenorhabditis* Genetics Center at the University of Minnesota. Animals were maintained on standard nematode growth medium plates containing 1 M CaCl_2_, 1 M MgS0_4_, 1 M KP0_4_, cholesterol, and spotted with standard *Escherichia coli* OP50 or *E. coli* OP50 with 10 μM 3TC. Live worms were immobilized in 1% 1‐phenoxy‐2‐propanol and then imaged using an EVOS M7000 fluorescence microscope (ThermoFisher) with a 40× objective. Approximately 16‐plane Z‐stacks were captured per animal, and images were deconvolved before analyzing dendrite degeneration, characterized by bead‐like structures that were counted using Celleste (ThermoFisher) imaging software. Lifespan experiments were performed using a Nemalife Infinity apparatus (NemaLife Inc.), a microfluidic device that allows for semi‐automated lifespan determination without the need for simultaneous pharmacological inhibition of progeny, as recently reported (Rahman et al., 2020). Approximately 70–80 N2 (wild‐type) adult worms were loaded into microfluidic chips containing standard liquid nematode growth medium. Chips were washed daily with S‐basal buffer to remove eggs/offspring, followed by the addition of fresh food (20 mg of *E. coli* OP50 per 1 mL of liquid growth medium) with or without 10 μM 3TC. Worms were video imaged daily, and live worms were counted by two blinded experimenters.

### 4.10 Human brain transcriptome analyses

Human TE/transcriptome data were generated from existing RNA‐seq datasets (North American Brain Expression Consortium [NABEC] and Religious Orders Study/Memory Aging Project [ROSMAP]). Data from subjects ≥25 years of age (when brain development is reported to end) in the NABEC dataset and all available raw fastqs on from ROSMAP subjects on the Synapse server were used for all analyses. TE transcripts and gene expression were analyzed following the same procedures described above for mouse RNA‐seq data but with the hg38 genome build, annotation and repeatmasker files, and differential expression analyses were performed using DESeq2 and all available samples in binary group comparisons (e.g., older vs. younger subjects and Alzheimer's disease vs. non‐cognitively impaired controls) as described in results sections and figure legends. Gene Ontology and KEGG pathway analyses were performed using g:Profiler as described above (Raudvere et al., 2019).

### 4.11 Statistics

Exact numbers of animals and subjects are reported in the figure legends, along with the relevant statistical analyses. Outlier analyses were performed using Grubbs' test, but no individual samples were identified as consistent outliers (i.e., in more than one comparison), so all data were used in analyses. As described above, differential expression of TE transcripts and genes was assessed using DESeq2, and significant differences were determined using the default Wald test. Correlations among TEs and age in human subjects and heatmaps of increased/decreased genes were analyzed using GraphPad Prism software (Pearson correlations on DESeq2 normalized counts). TE comparisons by type were performed using Shapiro–Wilk normality testing followed by Mann–Whitney test versus the mean Log2 fold‐change for all transcripts with >10 mean counts (to account for any global transcriptome, library preparation and/or normalization artifacts). For overlap comparisons of TEs in the NABEC and ROSMAP datasets, the most highly expressed TEs in subjects with higher versus lower cognitive function (Mini Mental State Exam scores), higher versus lower pathology (Braak staging) and Alzheimer's disease versus cognitively normal controls were identified as those with Log2 fold‐change greater than the mean Log2 fold‐change for all TEs. All human and mouse Gene Ontology/KEGG pathway analyses were performed using g:Profiler *p*‐value outputs (Raudvere et al., 2019). In mouse experiments, differences among groups (including Novel Object Recognition and Elevated Plus Maze, immunoblotting and ELISAs) were determined using a one‐way ANOVA with a Tukey's multiple comparison post hoc test. Barnes Maze data were analyzed using a 2 × 2 mixed effects (day × treatment) repeated measures ANOVA. Metabolomics heatmaps and partial least squares‐discriminant analyses were performed using the top 50 most significantly different metabolites determined by Log2 fold‐change. Differences in dendrite beading were measured using Student's *t*‐test, and *C. elegans* lifespan/survival differences were determined using a Log‐rank (Mantel‐Cox) statistics test.

## AUTHOR CONTRIBUTIONS

D.W. designed the study, wrote the paper, generated/analyzed human and mouse data, conducted the mouse study/behavioral experiments, and performed *C. elegans* experiments. M.E.S., C.M.M., and A.N.C. analyzed and generated human transcriptome data. S.C.O. performed immunoblots. S.D.B. and R.A.G. generated/analyzed *C. elegans* data. D.N. generated/analyzed metabolomics data. D.S.L. assisted with mouse experiments and metabolomics, wrote/edited corresponding parts of the paper, and provided conceptual insight. C.D.L. provided conceptual insight, data analysis and editing suggestions. T.J.L. designed the study, wrote/edited the paper, assisted with human and mouse transcriptome analyses, provided conceptual insight, and provided funding.

## CONFLICT OF INTEREST STATEMENT

The authors declare no conflicts of interests.

## Supporting information


**Supplementary Figure 1** Supporting data/information for Figures 1 and 3 (mouse 3TC treatment and RNA‐seq analyses). (a) Food and water consumption (g/mouse/day) in control and 3TC treated mice. (b) MA plot showing all changes in genes and TE transcripts in older versus younger mice.
**Supplementary Figure 2** Supporting data/information for Figure 2 (mouse hippocampus transcriptome analyses). (a–c) KEGG pathway analyses of increased/decreased gene expression signatures in older, younger and older 3TC‐treated mice.
**Supplementary Figure 3** Supporting data/information for Figure 3 (mouse brain immunoblot, ELISA and metabolomics analyses). (a) Western blot analyses of phosphorylated cGAS and TBK1, as well as Iba1 in younger, older and older 3TC‐treated mice. (b) ELISA analyses of IFN‐β in cortex and plasma concentrations of TNF‐α, IL‐1β, IL‐6, and IFN‐ɣ in younger, older and older 3TC‐treated mice. (c) Pathway analysis (MetaboAnalyst) of metabolic pathways most impacted by aging and 3TC treatment in older mice.
**Supplementary Figure 4** Supporting data/information for Figure 6 (human brain RNA‐seq analyses). (a) MA plot all age‐related changes in genes and TE transcripts in 10 older versus 10 younger (age/sex‐matched) subjects in NABEC dataset. (b, c) KEGG pathway analyses of gene expression signatures in the same subjects. (d) MA plot showing all changes in genes and TE transcripts in age/sex‐matched Alzheimer’s disease versus cognitively normal subjects in the ROSMAP dataset. (e, f) KEGG pathway analyses of increased/decreased gene expression signatures in the same subjects.

**Appendix S1:** Supporting Information

## Data Availability

All raw data that support transcriptome findings are publicly available. Human Alzheimer's disease and brain aging datasets: ROSMAP (Synapse server, accession ID syn3219045), NABEC (dbGaP server, accession ID phs001354.v1.p1). RNA‐seq data generated as part of this study are published on the Gene Expression Omnibus (GEO) server (GSE213848).
