# Supplementary material for: The reverse transcriptase inhibitor 3TC protects against age‐related cognitive dysfunction
Source: Aging Cell. 2023 Mar 22;22(5):e13798. doi: 10.1111/acel.13798 (PMC10186603; doi:10.1111/acel.13798)
Supplement: Supplementary file 1 — Supplementary Figure 1 Supporting data/information for Figures 1 and 3 (mouse 3TC treatment and RNA‐seq analyses). (a) Food and water consumption (g/mouse/day) in control and 3TC treated mice. (b) MA plot showing all changes in genes and TE transcripts in older versus younger mice. Supplementary Figure 2 Supporting data/information for Figure 2 (mouse hippocampus transcriptome analyses). (a–c) KEGG pathway analyses of increased/decreased gene expression signatures in older, younger and older 3TC‐treated mice. Supplementary Figure 3 Supporting data/information for Figure 3 (mouse brain immunoblot, ELISA and metabolomics analyses). (a) Western blot analyses of phosphorylated cGAS and TBK1, as well as Iba1 in younger, older and older 3TC‐treated mice. (b) ELISA analyses of IFN‐β in cortex and plasma concentrations of TNF‐α, IL‐1β, IL‐6, and IFN‐ɣ in younger, older and older 3TC‐treated mice. (c) Pathway analysis (MetaboAnalyst) of metabolic pathways most impacted by aging and 3TC treatment in older mice. Supplementary Figure 4 Supporting data/information for Figure 6 (human brain RNA‐seq analyses). (a) MA plot all age‐related changes in genes and TE transcripts in 10 older versus 10 younger (age/sex‐matched) subjects in NABEC dataset. (b, c) KEGG pathway analyses of gene expression signatures in the same subjects. (d) MA plot showing all changes in genes and TE transcripts in age/sex‐matched Alzheimer’s disease versus cognitively normal subjects in the ROSMAP dataset. (e, f) KEGG pathway analyses of increased/decreased gene expression signatures in the same subjects. [file ACEL-22-e13798-s002.docx]

**Supplementary Figures and Legends**

**
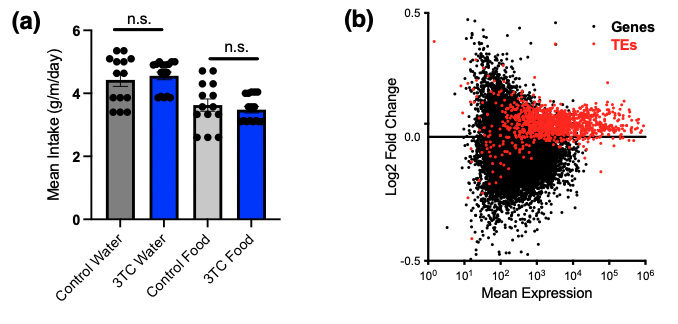
**

**Supplementary Figure 1. Supporting data/information for Figures 1 and 3 (mouse 3TC treatment and RNA-seq analyses). (a)** Food and water consumption (g/mouse/day) in control and 3TC treated mice**. (b)** MA plot showing all changes in genes and TE transcripts in older vs. younger mice.


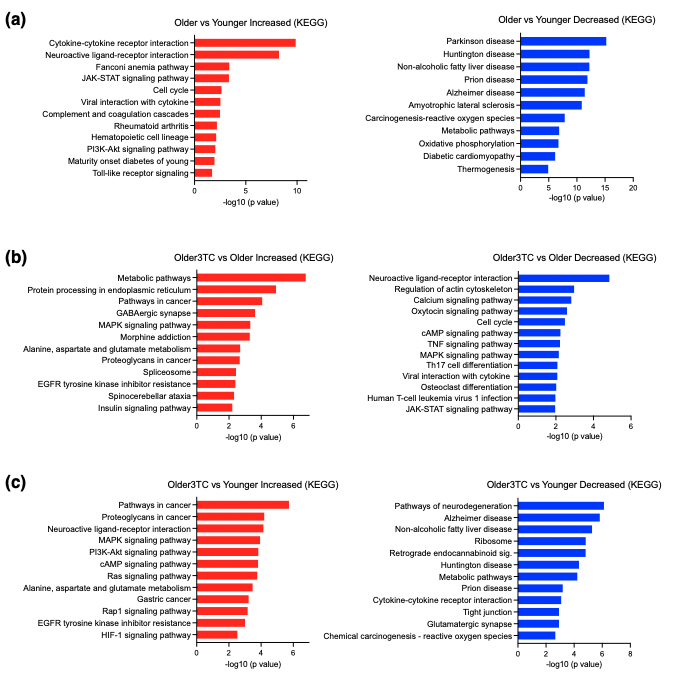


**Supplementary Figure 2. Supporting data/information for Figure 2 (mouse hippocampus transcriptome analyses). (a-c)** KEGG pathway analyses of increased/decreased gene expression signatures in older, younger and older 3TC-treated mice.

**
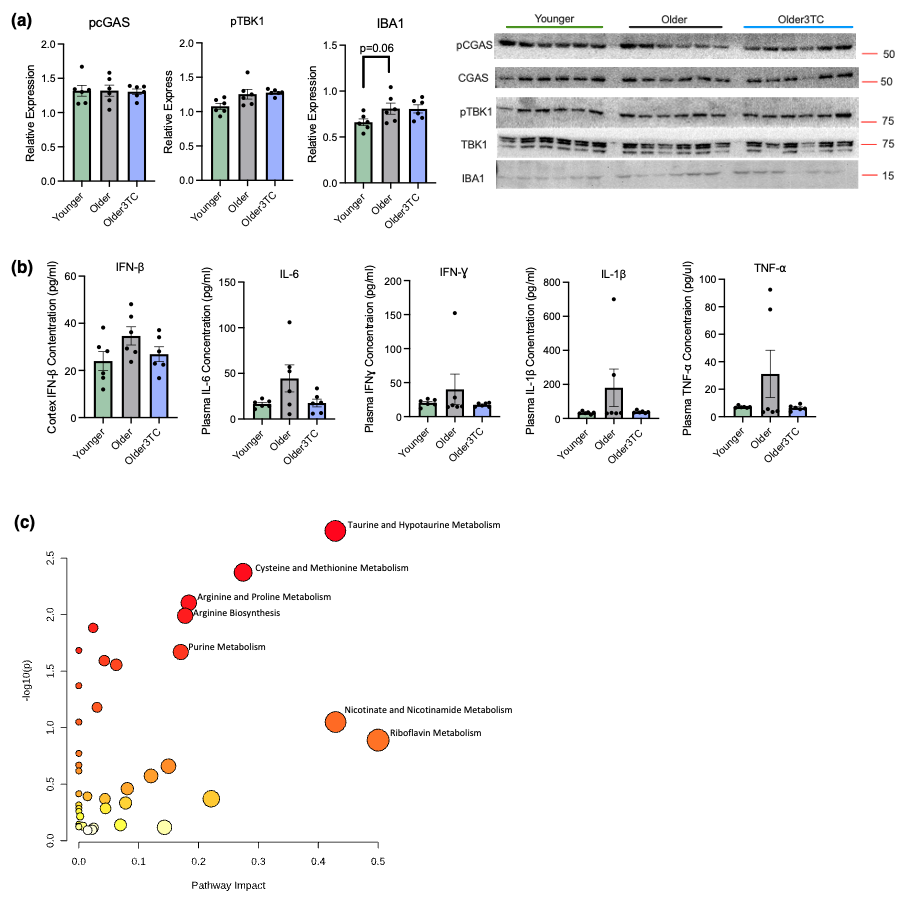
**

**Supplementary Figure 3. Supporting data/information for Figure 3 (mouse brain immunoblot, ELISA and metabolomics analyses). (a)** Western blot analyses of phosphorylated cGAS and TBK1, as well as Iba1 in younger, older and older 3TC-treated mice. **(b)** ELISA analyses of IFN-β in cortex and plasma concentrations of TNF-α, IL-1β, IL-6, and IFN-ɣ in younger, older and older 3TC-treated mice. **(c)** Pathway analysis (MetaboAnalyst) of metabolic pathways most impacted by aging and 3TC treatment in older mice.

**
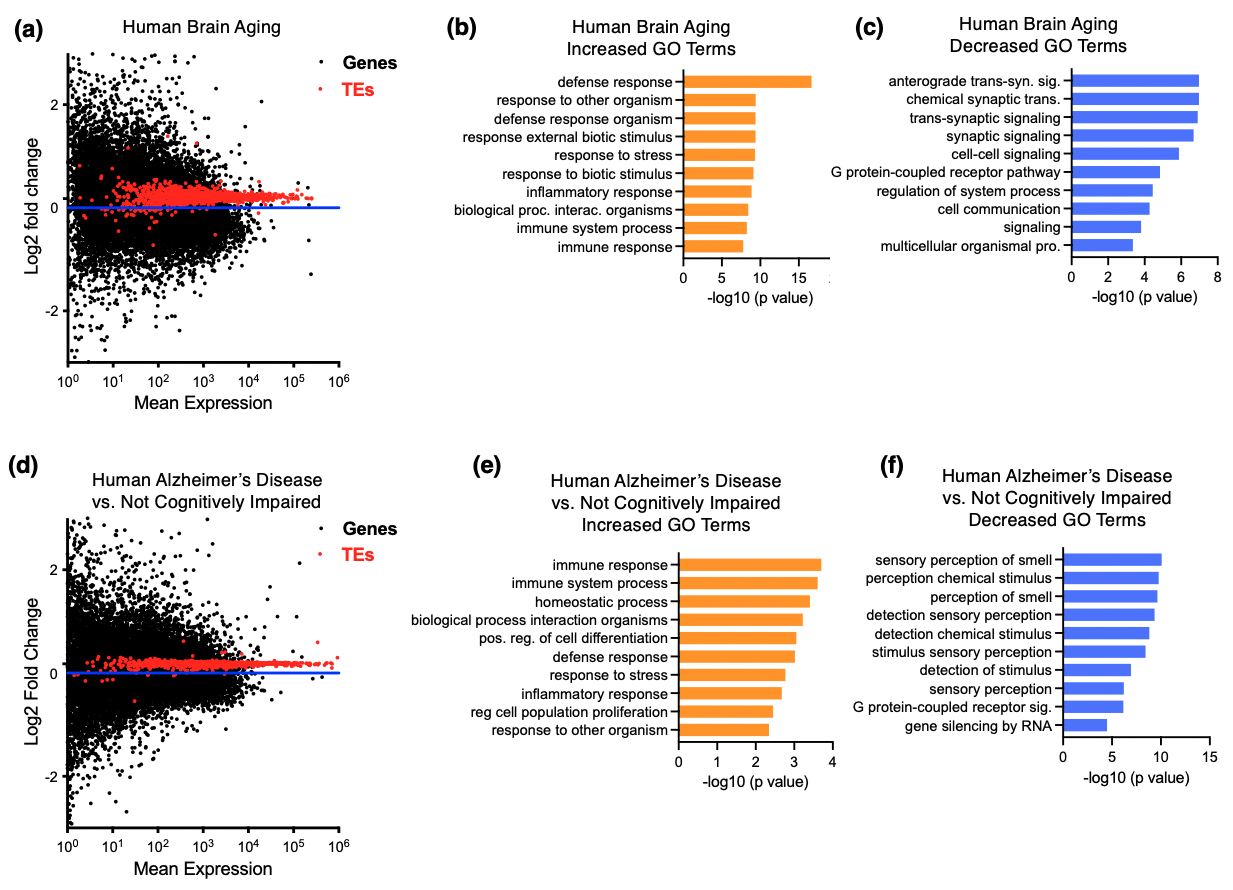
**

**Supplementary Figure 4. Supporting data/information for Figure 6 (human brain RNA-seq analyses). (a)** MA plot all age-related changes in genes and TE transcripts in 10 older vs. 10 younger (age/sex-matched) subjects in NABEC dataset. **(b,c)** KEGG pathway analyses of gene expression signatures in the same subjects. **(d)** MA plot showing all changes in genes and TE transcripts in age/sex-matched Alzheimer’s disease vs. cognitively normal subjects in the ROSMAP dataset. **(e,f)** KEGG pathway analyses of increased/decreased gene expression signatures in the same subjects.
